# Effect of pellet die thickness on standardized ileal digestibility of amino acids when pelleting diets fed to growing pigs

**DOI:** 10.1093/tas/txaf163

**Published:** 2025-12-13

**Authors:** Diego A Lopez, Matt D Miesner, Jordan T Gebhardt, Charles R Stark, Hans H Stein, Chad B Paulk

**Affiliations:** Department of Grain Science, Kansas State University, Manhattan, KS 66506, United States; Department of Diagnostic Medicine/Pathobiology, College of Veterinary Medicine, Kansas State University, Manhattan, KS 66506, United States; Department of Diagnostic Medicine/Pathobiology, College of Veterinary Medicine, Kansas State University, Manhattan, KS 66506, United States; Department of Grain Science, Kansas State University, Manhattan, KS 66506, United States; Department of Animal Sciences, University of Illinois, Urbana, IL 61801, United States; Department of Grain Science, Kansas State University, Manhattan, KS 66506, United States

**Keywords:** amino acids, digestibility, growing pigs, pellet die thickness, pelleting

## Abstract

Components of the pelleting process, such as steam conditioning and feed retention time in the conditioner and die, expose feed to various degrees of heat, moisture, pressure, and shear which changes its physical and chemical characteristics. These changes may influence nutrient digestibility. Therefore, the objective of this experiment was to determine the effect of different pellet die thicknesses on the apparent ileal digestibility (**AID**) and standardized ileal digestibility (**SID**) of crude protein (**CP**) and amino acids (**AA**) in commercial diets fed to pigs. A total of 12 growing barrows with an initial average body weight of 77.1 ± 3.53 kg were allotted to a triplicated 4 × 4 Latin square design with 4 treatments and 4 periods for a total of 12 replicate pigs per treatment. The dietary treatments consisted of a mash diet and three separate diets that were pelleted using a 1-ton pellet mill equipped with different pellet die length: diameter ratios (**L:D**) of 6 (26.5- × 4.4-mm), 8 (35.2- × 4.4-mm), and 10 (44.0- × 4.4-mm). The pelleted diets were steam conditioned for 30 s (Wenger twin staff pre-conditioner, Model 150), production rate was kept constant at 708 kg per hour, and conditioning temperature was adjusted to accomplish hot pellet temperature of 85°C. Ileal cannulated pigs were housed individually in pens equipped with a feeder, a drinker, smooth-side walls, and a fully slatted metal floor. For the AID and SID of CP and AA, the diets pelleted using the L:D 8 or L:D 10 pellet dies were greater (*P* < 0.05) than the mash diet and the diet pelleted using the L:D 6 pellet die, except for Lys, Met, Trp, and Gly. Diets pelleted using an L:D 10 pellet die had increased (*P* < 0.05) SID Lys compared with the mash control with other treatments being intermediate. Diets pelleted with the L:D 8 or L:D 10 die had increased (*P* < 0.05) SID of Met compared with the mash diet, whereas there was no difference in SID of Met between the mash control and L:D 6 and L:D 8 pelleted diets. For Trp and Gly, the AID and SID of the diet pelleted using the L:D 8 pellet die were greater (*P* < 0.05) than if the L:D 6 die was used with the other treatments being intermediate. Results indicate that AA digestibility improvement resulting from pelleting diets depends on pellet die thickness with diets pelleted using an L:D of 8 or 10 having improved AA digestibility for specific AA.

## Teaser

Diets pelleted with thicker dies had improved digestibility of crude protein and amino acids.

## Introduction

Key components of the pelleting process, such as steam conditioning and the time feed remains in the conditioner and die, subject feed to varying levels of heat, moisture, pressure, and shear. These conditions alter the physical and chemical properties of the feed. During steam conditioning and transit of feed through the die, heat and moisture plasticize the soluble fractions of the diet, promoting agglomeration of dietary components (Lundblad et al. 2009). Pelleting is widely used in swine nutrition and feeding to enhance nutrient utilization, feed efficiency, handling characteristics, and bulk density (Vukmirović et al. 2017). The primary improvement in feed efficiency for swine is often attributed to reduced feed wastage (Nemechek et al. 2015).

While reduced feed wastage is a major factor in improved feed efficiency, there is ongoing discussion about how much of the benefit from pelleting is due to enhanced nutrient digestibility. This has led to interest in optimizing specific pelleting parameters and settings, such as conditioning temperature, moisture addition, and die specification, to not only improve pellet quality but also enhance the nutritional value of the feed (Svihus and Zimonja 2011; Abdollahi et al. 2013; Rojas et al. 2016; Rojas and Stein 2017). Among these parameters, pellet die specifications play a critical role in determining the friction, heat, and pressure exerted on the feed during pellet formation (Behnke 1994; Knarr et al. 2024a). A key aspect of die specification is pellet die thickness, which is defined as the distance from the entry to the exit of each die hole and is commonly expressed as part of the length-to-diameter ratio (**L:D**), which also includes the hole diameter (Schofield and American Feed Industry Association 2005).

Pelleting improves nutrient digestibility, particularly crude protein (**CP**) and amino acids (**AA**), largely due to protein denaturation and inactivation of antinutritional factors during processing (Rojas et al. 2016; Lee et al. 2025; Dunmire et al. 2024). However, these benefits can be counterbalanced by the risk of heat damage, which may reduce the availability and digestibility of key nutrients if processing parameters become excessive (González-Vega et al. 2011). One factor contributing to increased temperature, pressure, and shear is the use of thicker pellet dies, which extend feed retention time within the die. This increased residence time enhances pellet quality but also elevates the temperature of pellets as they exit the die (Saensukjaroenphon 2019). Whereas thicker dies improve physical pellet quality (Saensukjaroenphon 2019; Truelock et al. 2020), it remains unclear if these same conditions positively or negatively impact nutrient digestibility. To the authors’ knowledge, no experiments have specifically determined effects of thicker pellet dies on nutrient digestibility in diets for pigs. Therefore, the objective of this experiment was to test the hypothesis that increased pellet die thickness will increase ileal digestibility of CP and AA by growing pigs.

## Materials and methods

The Institutional Animal Care and Use Committee at Kansas State University approved the use of animals for this experiment. Diets were mixed and processed at the Kansas State University O. H. Kruse Feed Technology and Innovation Center (Manhattan, KS).

### Dietary treatments

Dietary treatments included a mash control, and three pelleted diets produced using pellet dies that varied in thickness, resulting in a total of four dietary treatments in the experiment. Pellet die diameters were held consistent (4.4-mm); therefore, treatments will be expressed based on the pellet die L:D, as L:D 6, L:D 8, and L:D 10. A corn, soybean meal, and distillers dried grains with solubles-based diet was formulated (Table 1) to meet or exceed the nutrient requirements of pigs between 75 and 100 kg (NRC 2012). Three batches of 726 kg and a fourth batch of 318 kg were mixed using a twin-shaft counterpoise mixer (Hayes and Stolz, model TRDB63-0152, Fort Worth, TX, USA) for a total of 2495 kg. The mixing time was 60 s for dry ingredients and 120 s after liquid ingredients were added. The 318 kg-batch was kept as mash to be the control treatment. The remaining three batches were mixed, stored in a bin together, and used for further processing.

**Table 1.** Ingredient and calculated nutrient composition of experimental diet (as-fed basis).^a^

Item	Basal diet
**Ingredient, %**	
** Corn**	60.84
** Soybean meal**	15.00
** Distillers dried grains with solubles**	20.00
** Soybean oil**	1.00
** Calcium carbonate**	1.05
** Monocalcium phosphate, 21% P**	0.50
** Sodium chloride**	0.40
** L-Lysine-HCl**	0.30
** Trace mineral premix^b^**	0.15
** Vitamin premix^c^**	0.25
** Phytase^d^**	0.01
** Titanium dioxide**	0.50
** Total**	100.00
**Calculated composition**	
** Metabolizable energy, kcal/kg**	3337
** Crude protein, %**	17.9
** Crude fat, %**	5.1
** Crude fiber, %**	3.6
** Ca, %**	0.65
** STTD P, %**	0.41
** Standardized ileal digestible AA, %**	…
** Lysine**	0.85
** Isoleucine:lysine**	69
** Leucine:lysine**	180
** Methionine:lysine**	32
** Methionine and cysteine:lysine**	61
** Threonine:lysine**	60
** Tryptophan:lysine**	17.4
** Valine:lysine**	81
** Histidine:lysine**	48
** Total lysine, %**	1.01

a A basal diet was formulated and divided into 4 aliquots. One aliquot was used without further processing, and the remaining three aliquots were pelletized using different pellet dies (L:D 6, 8, and 10).

b Provided per kg of diet: 110 mg of Zn from zinc sulfate; 110 mg of Fe from iron sulfate; 33 mg of Mn from manganese oxide; 17 mg of Cu from copper sulfate; 0.30 mg of Se from sodium selenite; 0.30 mg of I from calcium iodate.

c Provided per kg of diet: 4134 IU vitamin A; 1653 IU vitamin D_3_; 44 IU vitamin E; 3 mg vitamin K; 0.03 mg vitamin B_12_; 50 mg of niacin; 28 mg pantothenic acid; 8 mg riboflavin.

d Quantum Blue 10 g (AB Vista, Marlborough, Wiltshire, U.K.) contained 10,000 FTU/g. At 0.01% inclusion, complete diets were expected to contain 500 FTU/kg with an estimated release of 0.10% STTD P.

### Feed processing

The experiment was designed to achieve pellet runs with a similar hot pellet temperature while using 3 different pellet dies. However, the increased thickness of the L:D 10 pellet die, resulted in increased hot pellet temperatures. Therefore, different conditioning temperatures were used to achieve similar hot pellet temperature across treatments. Pelleted diets were steam conditioned (25 × 140 cm Wenger twin staff pre-conditioner, Model 150, Wenger, Sabetha, KS, USA) for 30 s at 79.6, 79.2, or 68.0°C, and subsequently pelleted using a 1-ton pellet mill (1112-2, California Pellet Mill, Crawfordsville, IN, USA) equipped with a 26.5- × 4.4-mm (L:D 6), 35.0- × 4.4-mm (L:D 8), or 44.0- × 4.4-mm (L:D 10; Table 2) pellet die, respectively. Reducing the temperature of conditioning in the pellet run using the L:D 10 die reduced the amount of steam and in turn the amount of moisture available to penetrate the feed during this run. Greater pressure is needed to push feed through a longer channel when a thicker pellet die is used, which may potentially decrease production rate. However, by reducing the amount of steam and in turn the amount of moisture in the feed, it was possible to match the production rate of the pellet run using the L:D 10 die with the runs using the L:D 6 and L:D 9 dies. Therefore, under the conditions of this experiment, it was possible to maintain the production rate (11.8 kg/min) and hot pellet temperature (83.9 to 85.5°C; Table 3) across treatments, at the expense of changes in conditioning temperature. After 15 min of warmup, each dietary treatment was pelleted in a single run that took approximately 40 min. After a completed pellet run, the pellet die was changed, and the mill would be allowed to warm up again.

**Table 2.** Specifications of dies used during manufacturing of dietary treatments.^a^

	Pellet die length: diameter		
**Item**	6	8	10
**Die hole diameter, mm (D)**	4.4	4.4	4.4
**Die effective thickness, mm (L)**	26.5	35.0	44.0
**Internal die diameter, cm**	30.5	30.5	30.5
**Internal die width, cm**	5.7	5.7	5.7
**Internal die surface area, cm2^b^**	547.2	547.2	547.2
**Holes per cm^b^**	2.2	2.2	2.6
**Total die holes^c^**	1188	1188	1442

a Specification values were measured unless otherwise is indicated.

b Internal die diameter × Internal die width × π.

c Calculated according to Saensukjaroenphon (2019).

**Table 3.** Pellet mill parameters and subsequent pellet quality of pelleted diets.^a^^,^^b^

	Pellet die length: diameter		
**Item**	6	8	10
**Production rate, kg/min**	11.8	11.8	11.8
**Conditioning temperature, °C**	79.6	79.2	68.0
**Conditioning time, s**	30.0	30.0	30.0
**Hot pellet temperature, °C**	83.9	85.5	84.3
**Die retention, s^c^**	1.6	2.2	3.3
**Pellet durability index (PDI), %^d^**	50.1	86.8	93.1

a Treatments were steam conditioned using a Wenger twin staff pre-conditioner (Model 150, Wenger, Sabetha, KS, USA) and subsequently pelleted using a California Pellet Mill (model 1112-2, Crawfordsville, IN, USA).

b The values for production rate and conditioning temperatures, and hot pellet temperature are the averages of six measurements taken at evenly spaced intervals over the duration of the pellet run.

c Calculated according to Saensukjaroenphon (2019) using the die specifications listed in Table 2.

d Holmen NHP100 (Norfolk, U.K.) method for 30 s; Samples were analyzed in duplicated.

During each pelleting run, the temperature in the conditioner, production rate, and hot pellet temperature were monitored and recorded every 8 min (Table 3). To record hot pellet temperature, a sample of pellets was placed into a pre-warmed double-wall thermos equipped with a thermometer. Die retention time was calculated for each pellet run by determining the amount of material that can be held in the effective length of the diet, divided by the flow rate of the material during the pellet run, as described by Saensukjaroenphon (2019). Pellet samples were collected after cooling for 10 min in a research cooler, sifted with a U.S. No. 5 (3.9 mm) sieve to separate pellets from fines, and stored in commercial paper feed sacks. Pellet durability index (**PDI**) was analyzed in duplicate, and the results were averaged, following the Holmen forced-air method (Evans 2023) using a NHP 100 (TekPro Ltd, Norfolk, U.K.) pellet tester with each run lasting 30 s. For each dietary treatment, PDI was calculated using the following equation (S269.5; ASAE 2012):


$$\mathrm{PDI}\;\left(\%\right)=\frac{\mathrm{Weight}\;\mathrm{of}\;\mathrm{recovered}\;\mathrm{pellets}}{\mathrm{Intitial}\;\mathrm{weight}\;\mathrm{of}\;\mathrm{pellets}}\times 100$$

### Animals and feeding

Twelve barrows (average initial body weight: 77.1 ± 3.53 kg; DNA 241 × 600, DNA, Columbus, NE) were surgically equipped with a T-cannula in the distal ileum (Stein et al. 1998) and allotted to a triplicated 4 × 4 Latin square design (Kim and Stein 2009) with four periods of 7 d and 4 dietary treatments. Therefore, each diet was fed to 3 pigs in each period for a total of 12 replicate pigs per dietary treatment for the 4 periods. Feed allowance for the pigs was 3.0 times the maintenance requirement for metabolizable energy (ie 197 kcal/kg body weight^0.60^; NRC 2012) with free access to water throughout the experiment. The daily allowance of feed per day was divided into 2 equal meals that were provided to the animal at 0800 and 1400 h.

### Experimental procedures

The first 5 days of each period were considered adaptation to the diet (Adeola 2001), and ileal sample collection took place on days 6 and 7. During each collection day, the lid was removed from the cannulas, and a plastic bag was attached to the end of the cannula using a plastic cable tie. The digesta flowing into the bag was collected every 30 min or when the bag was full, for a total of 8 h each day. The samples collected were immediately stored at −20°C to prevent any bacterial degradation of AA or enzymatic activity.

### Data collection and sample analysis

Ileal digesta samples were thawed, subsampled, lyophilized, and ground prior analysis. Diet and ileal samples were analyzed for nitrogen using the combustion method (LECO analyzer; method 990.03; AOAC 2019) and CP was calculated as nitrogen × 6.25. Diet and ileal samples were also analyzed for dry matter (**DM**; method 930.15; AOAC 2019), AA concentrations (method 982.30 E [a, b, c]; AOAC 2019), and titanium concentrations (Myers et al. 2004). Diet samples were also analyzed for crude fat (method 920.39; AOAC 2019), crude fiber (method 978.10; AOAC 2019), and ash (method 942.05; AOAC 2019).

### Calculations and statistical analysis

Apparent ileal digestibility (**AID**) and standardized ileal digestibility (**SID**) of CP and AA were calculated for the 4 dietary treatments using the equations described by Stein et al. (2007), and the values for the basal endogenous losses of CP and AA were obtained from a previous experiment (Lopez 2025) conducted with the same pigs used in the present experiment.

Data were analyzed using the GLIMIX procedure of SAS (SAS Institute Inc., Cary, NC) using pig as the experimental unit and the random effects of pig and period and the fixed effect of dietary treatment. For each independent variable, least square means were calculated and then separated using the PDIFF option. Studentized residuals were used to remove outliers, for any value outside of more than ±3. Treatment differences were considered significant at *P* ≤ 0.05 and marginally significant at 0.05 < *P* ≤ 0.10.

## Results

The calculations of die retention time indicate that the feed spent 1.6, 2.2, or 3.3 s in the pellet die with L:D of 6, 8, and 10, respectively. Pellet durability index was analyzed for each dietary treatment, and the results indicate an increase in this measurement as the die thicknesses increased. The pellets manufactured using the die with L:D 6 had the lowest PDI (50.1%), followed by the L:D 8 (86.6%), and pellets produced using the L:D 10 die had the greatest PDI (93.1%). The differences among the analyzed nutrient composition of the diets (Table 4) were considered to be within the range of analytical or sampling variability.

**Table 4.** Analyzed composition of experimental diets (as-fed basis).^a^

		Pellet die length: diameter		
**Item, %**	Mash diet	6	8	10
**Dry matter**	89.92	88.57	87.77	87.82
**Crude protein**	18.33	18.45	17.67	18.16
**Crude fat**	4.20	4.92	4.72	4.79
**Crude fiber**	3.14	3.78	3.26	3.09
**Ash**	4.80	5.17	5.15	5.13
**Indispensable AA**				
** Arginine**	0.99	1.05	1.08	0.99
** Histidine**	0.49	0.50	0.51	0.48
** Isoleucine**	0.74	0.76	0.78	0.73
** Leucine**	1.85	1.77	1.86	1.79
** Lysine**	1.02	1.15	1.14	1.02
** Methionine**	0.33	0.33	0.33	0.31
** Phenylalanine**	0.90	0.91	0.94	0.88
** Threonine**	0.66	0.69	0.70	0.65
** Tryptophan**	0.16	0.16	0.17	0.15
** Valine**	0.88	0.92	0.93	0.87
**Dispensable AA**				
** Alanine**	1.12	1.09	1.13	1.09
** Aspartic acid**	1.50	1.61	1.64	1.53
** Cysteine**	0.36	0.36	0.37	0.35
** Glutamic acid**	3.24	3.22	3.33	3.18
** Glycine**	0.72	0.76	0.78	0.72
** Serine**	0.76	0.78	0.80	0.76
** Tyrosine**	0.65	0.65	0.68	0.65

a Samples were analyzed at the University of Missouri Agricultural Experiment Station Chemical Laboratories in Columbia, MO.

There was an overall treatment effect (*P *< 0.01) on AID of DM, CP, and all AA (Table 5). The AID of DM was greater (*P* < 0.05) in the diet pelleted using the L:D 8 or 10 die compared with the L:D 6 die, but AID of DM in the diet pelleted with the L:D 6 die was not different from the mash diet. No differences were observed between the mash diet and the diets pelleted using the L:D 6 or L:D 8 dies. Diets pelleted using the L:D 8 or the 10 dies had greater (*P* *<* 0.05) AID of CP and most AA, except for Lys, Met, Trp, and Gly, compared with the mash diet or the diet pelleted using the L:D 6 die. The diet pelleted using the L:D 10 die had greater (*P* < 0.05) AID of Met than the mash diet and the L:D 6 diet, whereas the diet pelleted using the L:D 8 die had greater (*P* < 0.05) AID of Met than the mash diet, but not the other pelleted diets. The diets pelleted using the L:D 8 and the L:D 10 dies had greater (*P* < 0.05) AID of Lys compared with the mash diet, but no differences were observed between diets pelleted using the L:D 6 die and those pelleted using the L:D 8 or the L:D 10 or mash diets. No differences between the mash diet and the pelleted diets were observed for AID of Trp, however, the AID of Trp was greater (*P* < 0.05) in the diet pelleted using the L:D 8 die compared with the L:D 6 die. Additionally, no differences were observed for the AID of Trp between the L:D 10 diet and either the L:D 6 or the L:D 8 diet. The AID of Gly was greater (*P* < 0.05) in the L:D 8 diet compared with the L:D 6 diet, however, no differences were observed between the mash diet and the pelleted diets using either the L:D 6 or the L:D 10 die.

**Table 5.** Apparent ileal digestibility (AID) of dry matter, crude protein and amino acid (AA) in dietary treatments^1^.

		Pellet die length: diameter				
**Item, %**	Mash	6	8	10	SEM	*P*
**Dry matter**	69.2^bc^	67.4^c^	70.2^ab^	72.2^a^	0.69	<0.001
**Crude protein**	75.4^b^	75.1^b^	77.4^a^	77.5^a^	0.73	<0.001
**Indispensable AA**						
** Arginine**	86.1^b^	87.0^b^	88.3^a^	88.2^a^	0.54	<0.001
** Histidine**	82.0^b^	82.2^b^	84.0^a^	84.0^a^	0.64	<0.001
** Isoleucine**	78.7^b^	79.8^b^	82.0^a^	82.0^a^	0.75	<0.001
** Leucine**	84.0^b^	84.3^b^	86.4^a^	86.9^a^	0.61	<0.001
** Lysine**	81.4^b^	82.3^ab^	83.8^a^	84.0^a^	1.55	<0.005
** Methionine**	84.3^c^	85.4^bc^	86.6^ab^	86.9^a^	0.61	<0.001
** Phenylalanine**	81.6^b^	82.4^b^	84.4^a^	84.6^a^	0.66	<0.001
** Threonine**	70.2^b^	70.8^b^	73.8^a^	73.7^a^	0.89	<0.001
** Tryptophan**	84.4^ab^	82.9^b^	85.9^a^	84.3^ab^	0.97	<0.012
** Valine**	76.0^b^	77.7^b^	79.9^a^	80.0^a^	0.79	<0.001
**Mean**	81.1^b^	81.8^b^	83.8^a^	83.9^a^	0.68	<0.001
**Dispensable AA**						
** Alanine**	79.3^b^	79.8^b^	82.3^a^	83.0^a^	0.73	<0.001
** Aspartic acid**	75.8^b^	76.8^b^	79.3^a^	79.0^a^	0.82	<0.001
** Cysteine**	70.7^b^	70.9^b^	73.8^a^	73.2^a^	1.00	<0.001
** Glutamic acid**	84.7^b^	84.4^b^	86.7^a^	86.7^a^	0.66	<0.001
** Glycine**	65.0^b^	64.3^b^	68.8^a^	66.6^ab^	1.39	<0.003
** Serine**	78.8^b^	79.3^b^	81.5^a^	82.0^a^	0.66	<0.001
** Tyrosine**	83.0^b^	83.5^b^	85.5^a^	86.1^a^	0.56	<0.001
**Mean**	79.3^b^	78.6^b^	81.3^a^	81.3^a^	0.82	<0.001
**Total AA**	80.1^b^	80.1^b^	82.4^a^	82.4^a^	0.75	<0.001

a–c Means within a row lacking a common superscript letter are different (*P <* 0.05).

1 N = 12.

The SID of CP was greater (*P* < 0.05) in the diets pelleted using the L:D 8 or L:D 10 dies, compared with the L:D 6 die, but no differences were observed between the mash diet and the diet pelleted using the L:D 6 or the L:D 8 dies (Table 6). Additionally, no differences were observed between the diet pelleted using the L:D 8 die and the L:D 10 die. The SID of most AA except Lys, Met, Trp, and Gly, were greater (*P* < 0.05) in the diets pelleted using the L:D 8 or the L:D 10 dies compared with the mash diet or the L:D 6 diet, but no differences were observed between the mash diet and the L:D 6 diet. The diet pelleted using the L:D 10 die had a greater (*P* < 0.05) SID of Lys than the mash diet, but there was no difference between the mash diet and the diets pelleted using the L:D 6 or the L:D 8 dies, or between the diet pelleted using the L:D 10 die and the diets pelleted using the L:D 6 or the L:D 8 dies. The SID of Met was less (*P* < 0.05) in the mash diet compared with the L:D 8 and the L:D 10 diets, but the SID of Met in the mash diet was no different from the L:D 6 diet. The SID of Met was also not different between the diet pelleted using the L:D 6 die and the diet pelleted using the L:D 8 die, or between the diet pelleted using the L:D 8 die and the diet pelleted using the L:D 10 die. For the SID of Trp and Gly, no differences were observed between the mash diet and the pelleted diets, regardless of the pellet die. However, the SID of Trp and Gly in the diet pelleted using the L:D 8 pellet die was greater (*P* < 0.05) than in the L:D 6 diet, but not different from the L:D 10 diet. No differences were observed in the SID of Trp and Gly between the diet pelleted using the L:D 6 die or the L:D 10 die.

**Table 6.** Standardized ileal digestibility of crude protein (CP) and AA in dietary treatments^1^^,^^2^.

		Pellet die length: diameter				
**Item, %**	Mash	6	8	10	SEM	*P*
**Crude protein**	83.0^bc^	82.5^c^	84.7^ab^	85.1^a^	0.73	<0.001
**Indispensable AA**						
** Arginine**	90.8^b^	91.3^b^	92.6^a^	92.8^a^	0.54	<0.001
** Histidine**	85.2^b^	85.3^b^	87.0^a^	87.2^a^	0.64	<0.001
** Isoleucine**	82.5^b^	83.4^b^	85.5^a^	85.7^a^	0.75	<0.001
** Leucine**	86.4^b^	86.7^b^	88.6^a^	89.2^a^	0.61	<0.001
** Lysine**	84.8^b^	85.3^ab^	86.8^ab^	87.4^a^	1.55	<0.006
** Methionine**	86.6^c^	87.7^bc^	88.8^ab^	89.3^a^	0.61	<0.001
** Phenylalanine**	84.6^b^	85.3^b^	87.3^a^	87.6^a^	0.66	<0.001
** Threonine**	77.2^b^	77.4^b^	80.2^a^	80.7^a^	0.89	<0.001
** Tryptophan**	91.0^ab^	89.4^b^	92.1^a^	91.3^ab^	0.97	<0.031
** Valine**	80.4^b^	81.8^b^	83.9^a^	84.3^a^	0.79	<0.001
**Mean**	84.8^b^	85.3^b^	87.2^a^	87.6^a^	0.68	<0.001
**Dispensable AA**						
** Alanine**	83.6^b^	84.2^b^	86.4^a^	87.3^a^	0.73	<0.001
** Aspartic acid**	80.4^b^	81.1^b^	83.4^a^	83.4^a^	0.82	<0.001
** Cysteine**	75.1^b^	75.2^b^	78.0^a^	77.7^a^	1.00	<0.002
** Glutamic acid**	87.3^b^	87.0^b^	89.1^a^	89.2^a^	0.66	<0.001
** Glycine**	83.9^ab^	82.0^b^	85.9^a^	85.1^ab^	1.39	<0.014
** Serine**	84.5^b^	84.7^b^	86.8^a^	87.6^a^	0.66	<0.001
** Tyrosine**	86.3^b^	86.7^b^	88.6^a^	89.4^a^	0.56	<0.001
**Mean**	88.3^bc^	87.4^c^	89.8^ab^	90.2^a^	0.82	<0.001
**Total AA**	86.7^b^	86.5^b^	88.6^a^	89.0^a^	0.75	<0.001

a–c Means within a row lacking a common superscript letter are different (*P* < 0.05).

1 N = 12.

2 Values for standardized ileal digestibility were obtained with the correction of apparent ileal digestibility values for basal endogenous losses of each individual AA and CP. These values were obtained from previously conducted research at the Swine Teaching and Research Center (STRC) at Kansas State University (g/kg of dry matter intake): Crude protein, 15.41; Arg, 0.52; His, 0.17; Ile 0.31; Leu, 0.48; Lys, 0.39; Met, 0.08; Phe, 0.30; Thr, 0.51; Trp, 0.12; Val, 0.42; Ala, 0.53; Asp 0.77; Cys, 0.18; Glu, 0.92; Gly, 1.52; Ser, 0.48; Tyr, 0.24

## Discussion

The pelleting parameters were selected for each pellet die treatment to balance production rate and hot pellet temperature across treatments. Production rate was standardized across the pellet runs to reduce possible variability that could confound the effect of the thickness of the pellet die. Changes in the residence time in the pellet die can be attributed to the effective length of the die, rather than changes in throughput or differences in conditioning time. Targeting similar hot pellet temperatures instead of similar conditioning temperatures decreased the possibility of heat damage or enzyme degradation in the pellets, which could confound the response in digestibility observed during the experiment. Hot pellet temperature is a better indicator for phytase degradation than conditioning temperature alone although reducing conditioning temperature reduced the amount of moisture in the feed during the pellet run using the L:D 10 die (Truelock et al. 2022). Thicker pellet dies require more pressure and friction for feed to pass through, but excess moisture can contribute to the formation of a thicker feed pad, which in turn can cause roll slips and die chokes (Evans 2023).

Increasing die L:D from 5.6 to 8.0 improves pellet durability but increases pellet mill energy consumption (Truelock et al. 2020). Behnke (2014) described the same positive correlation between die L:D and pellet durability, which can be attributed to the increased pressure and resistance generated by a larger die L:D. When die hole diameter remains constant, a pellet die with a larger die L:D is thicker than a die with a smaller L:D. Thus, feed retention within the die is longer with a thicker die and is a primary factor in determining pellet durability. This is due to increased exposure to heat and pressure that leads to the physicochemical reactions experienced during pelleting (Bastiaansen et al. 2024), which enhance binding among particles and contribute to improved pellet durability (Saensukjaroenphon 2019; Truelock et al. 2020; Knarr et al. 2024a). Therefore, the observation that increased pellet die thickness in the present experiment resulted in greater PDI is in agreement with previous data. The magnitude of increase in PDI as L:D increased from 6 to 8 is greater than the increase from L:D 8 to 10. This observation indicates that the pressure and friction as the feed went through the L:D 6 die was not enough to promote the physicochemical reactions that result in a more structurally sound pellet. Furthermore, the retention time in the pellet die was considerably shorter in the L:D 6 pellet die, compared with the L:D 8 and L:D 10. The time of residence along with the pressure, friction, and higher temperatures during the pellet runs using the L:D 8 and 10 pellet dies promoted the physicochemical changes to a greater degree than the L:D 6 die, which resulted in the increase in AA digestibility that was observed.

During pelleting, proteins are exposed to heat and mechanical forces that disrupt their tertiary structure, leading to irreversible denaturation and potential AA binding. In general, heat treatment improves protein digestibility by inactivating antinutritional factors such as enzyme inhibitors and unfolding proteins to enhance enzymatic access (Hancock and Behnke 2001; Svihus and Zimonja 2011; Lancheros et al. 2020). However, it is not known if this effect is a result of denaturation of the proteins that become more available for digestion and absorption, or of elimination of antinutritional factors (Svihus and Zimonja 2011). The combination of high temperature, shear force, and moisture during pelleting can potentially promote the Maillard reaction. This reaction involves the interaction of free amine groups in AA and proteins with the carbonyl groups of reducing sugars (e.g., glucose, galactose, and fructose), which can reduce the availability and utilization of AA (Thorpe and Baynes 2003; Pahm et al. 2008; González-Vega et al. 2011; Peng et al. 2011). However, digestibility of CP and AA in swine diets is usually increased by pelleting (Rojas et al. 2016; Dunmire et al. 2024; Lee et al. 2025) indicating that formation of Maillard reaction products during pelleting likely is not a concern. Indeed, pelleting diets with different concentrations of reducing sugar and free AA did not induce Maillard reactions and improved SID of AA further indicating that pelleting diets at less than 90°C has no negative impact on AA digestibility (Dunmire et al. 2024).

Because thicker pellet dies increase feed retention time, thicker dies expose feed to greater heat and pressure, which promotes physicochemical reactions (Bastiaansen et al. 2024). The observation that pelleting diets using the L:D 8 and 10 improved ileal AA digestibility is in agreement with reported data (Rojas et al. 2016; Dunmire et al. 2024; Lee et al. 2025). However, because no differences were observed between the diet pelleted using the L:D 6 die and the mash diet, it is hypothesized that diets pelleted using the L:D 6 die did not undergo the necessary physiochemical changes to result in improvements of nutrient digestibility. This is also demonstrated by the much poorer PDI achieved when pelleting diets using the L:D 6 die instead of the L:D 8 or the L:D 10 die.

To our knowledge, effects of pellet die thicknesses on ileal digestibility of nutrients in diets fed to growing pigs have not been determined. However, the AID of AA by broiler chickens is greater in diets pelleted using a pellet die with an L:D of 10 compared with a L:D 7.1 die (Knarr et al. 2024b). A longer retention time in the die allows friction and pressure to increase the temperature of the feed, and simultaneously allows more time for the moisture to diffuse into the feed which as mentioned before, is a slower process than heat diffusion (Thomas and van der Poel 2020), potentially decreasing the threshold of temperature needed for protein denaturation and inactivation of anti-nutritional factors. Therefore, it is likely that it is the combination of both the elevated temperature in diets pelleted using the thicker dies and the increased moisture that resulted in the increased SID of AA that were observed.

In conclusion, pelleting diets using L:D 8 or L:D 10 pellet dies resulted in greater AID and SID of CP and most AA compared with a mash dietary treatment. However, no differences were observed between the diet pelleted using the L:D 6 die and the mash diet. These results indicate that the magnitude of the forces exerted on the feed during pelleting (ie temperature, pressure, friction) can influence the degree of the positive response observed from pelleting on CP and AA digestibility.

## List of abbreviations:

AA: amino acid
AID: apparent ileal digestibility
CP: crude protein
DM: dry matter
L:D: length to diameter ratio
PDI: pellet durability index
SID: standardized ileal digestibility

## References

[txaf163-B1] Abdollahi M. R. , Ravindran V., Svihus B. 2013. Pelleting of broiler diets: an overview with emphasis on pellet quality and nutritional value. Anim. Feed Sci. Technol. 179:1–23. 10.1016/j.anifeedsci.2012.10.011

[txaf163-B2] Adeola O. 2001. Digestion and balance techniques in pigs. In: Lewis A. J., Southern L. L., editors, Swine nutrition. CRC Press, Washington, DC. p. 903–916.

[txaf163-B3] AOAC. 2019. Official methods of analysis of AOAC international. 21st ed. AOAC International, Rockville, MD.

[txaf163-B4] ASAE. 2012. Densified products for bulk handling—definitions and method. ASAE standard S269.5. ASABE. St Joseph, MI.

[txaf163-B5] Bastiaansen T. M. M. et al. 2024. Separating the effect of die geometry and mash residence time in the die on biomass and livestock feed pellet manufacturing. Biomass Bioenergy. 190:107383. 10.1016/j.biombioe.2024.107383

[txaf163-B6] Behnke K. C. 1994. Processing factors influencing pellet quality. Kansas State University, Manhattan, KS. https://www.adiveter.com/ftp_public/articulo1477.pdf

[txaf163-B7] Behnke K. C. 2014. Section 5: Pellet durability, Chapter 19: Factors affecting pellet quality. In: Feed pelleting reference guide. WATT Global Media and Kansas State University. https://img.feedstrategy.com/files/base/wattglobalmedia/all/image/static/feedstrategy/feed-pelleting-reference-guide/5-19_Factors_affecting_pellet_quality.pdf

[txaf163-B8] Dunmire K. M. et al. 2024. Effect of the pelleting process on diet formulations with varying levels of crystalline amino acids and reducing sugars on digestibility in growing pigs. J. Anim. Sci. 102:1–9. 10.1093/jas/skad423 PMC10889723 38170568

[txaf163-B9] Evans C. E. 2023. Evaluation of feed processing techniques on feed quality and monogastric nutrition. [PhD dissertation]. Kansas State University.

[txaf163-B10] González-Vega J. C. , Kim B. G., Htoo J. K., Lemme A., Stein H. H. 2011. Amino acid digestibility in heated soybean meal fed to growing pigs. J. Anim. Sci. 89:3617–3625. 10.2527/jas.2010-3465 21742940

[txaf163-B11] Hancock J. D. , Behnke K. C. 2001. Use of ingredient and diet processing technologies (grinding, mixing, pelleting, and extruding) to produce quality feeds for pigs. In: Lewis A. J., Southern L. L., editors, Swine nutrition. CRC Press, Washington, DC.. p. 473–501.

[txaf163-B12] Kim B. G. , Stein H. H. 2009. A spreadsheet program for making a balanced Latin square design. Rev. Colomb. Cienc. Pecu. 22:6–596. 10.17533/udea.rccp.324493

[txaf163-B13] Knarr L. E. , Bowen K. M., Ferrel J., Moritz J. S. 2024a. Azomite, dacitic (rhyolitic) tuff breccia, included at 0.25% in feed manufactured with 32, 38, and 45 mm pellet die thicknesses increased pellet production rate by 5.0, 7.9, and 11.8%, respectively. J. Appl. Poultry Res. 33:100389. 10.1016/j.japr.2023.100389

[txaf163-B14] Knarr L. E. et al. 2024b. Pellet die thickness and a commercial throughput agent interacted to demonstrate that high frictional heat increased apparent ileal amino acid digestibility, but did not influence trypsin inhibitor activity or male broiler performance. J. Appl. Poultry Res. 33:100460. 10.1016/j.japr.2024.100460

[txaf163-B15] Lancheros J. P. , Espinosa C. D., Stein H. H. 2020. Effects of particle size reduction, pelleting, and extrusion on the nutritional value of ingredients and diets fed to pigs: a review. Anim. Feed Sci. Technol. 268:114603. 10.1016/j.anifeedsci.2020.114603

[txaf163-B16] Lee S. A. , Paulk C. B., Stein H. H. 2025. Interactive effects of pelleting and particle size reduction of corn on ileal digestibility of starch, acid-hydrolyzed ether extract, and amino acids in corn-soybean meal diets fed to growing pigs. Anim. Feed Sci. Technol. 327:116437. 10.1016/j.anifeedsci.2025.116437

[txaf163-B17] Lopez D. A. 2025. Effect of feed technologies on the digestibility of energy and nutrients in diets fed to growing pigs. [Ph.D. dissertation]. Kansas State University.

[txaf163-B18] Lundblad K. K. et al. 2009. The effect of adding water into the mixer on pelleting efficiency and pellet quality in diets for finishing pigs without and with use of an expander. Anim. Feed Sci. Technol. 150:295–302. 10.1016/j.anifeedsci.2008.10.006

[txaf163-B19] Myers W. D. , Ludden P. A., Nayigihugu V., Hess B. W. 2004. Technical note: a procedure for the preparation and quantitative analysis of samples for titanium dioxide. J. Anim. Sci. 82:179–183. 10.2527/2004.821179x 14753360

[txaf163-B6505991] Nemechek J. E. , M. D. Tokach, S. S. Dritz, E. D. Fruge, E. L. Hansen, R. D. Goodband, J. M. DeRouchey, and J. C. Woodworth. 2015. Effects of diet form and feeder adjustment on growth performance of nursery and finishing pigs. J. Anim. Sci. 93:4172–4180. 10.2527/jas.2015-9028 26440197

[txaf163-B20] NRC. 2012. Nutrient requirements of swine. 11th rev. ed. Natl. Acad. Press, Washington, DC.

[txaf163-B21] Pahm A. A. , Pedersen C., Stein H. H. 2008. Application of the reactive lysine procedure to estimate lysine digestibility in distillers dried grains with solubles fed to growing pigs. J. Agric. Food Chem. 56:9441–9446. 10.1021/jf801618g 18817414

[txaf163-B22] Peng X. , Ma J., Chen F., Wang M. 2011. Naturally occurring inhibitors against the formation of advanced glycation end-products. Food Funct. 2:289–301. 10.1039/c1fo10034c 21779567

[txaf163-B23] Rojas O. J. , Vinyeta E., Stein H. H. 2016. Effects of pelleting, extrusion, or extrusion and pelleting on energy and nutrient digestibility in diets containing different levels of fiber and fed to growing pigs. J. Anim. Sci. 94:1951–1960. 10.2527/jas.2015-0137 27285693

[txaf163-B24] Rojas O. J. , Stein H. H. 2017. Processing of ingredients and diets and effects on nutritional value for pigs. J. Anim. Sci. Biotechnol. 8:48. 10.1186/s40104-017-0177-1 28572976 PMC5452379

[txaf163-B25] Saensukjaroenphon M. 2019. The effect of pelleting parameters on phytase stability and pellet quality. [PhD dissertation]. Kansas State University.

[txaf163-B26] Schofield E. K. and American Feed Industry Association. 2005. Feed manufacturing technology V. American Feed Industry Association. Arlington, VA.

[txaf163-B27] Stein H. H. , Sève B., Fuller M. F., Moughan P. J., de Lange C. F. M. 2007. Invited review: amino acid bioavailability and digestibility in pig feed ingredients: terminology and application. J. Anim. Sci. 85:172–180. 10.2527/jas.2005-742 17179553

[txaf163-B28] Stein H. H. , Shipley C. F., Easter R. A. 1998. Technical note: a technique for inserting a T-cannula into the distal ileum of pregnant sows. J. Anim. Sci. 76:1433–1436. 10.2527/1998.7651433x 9621950

[txaf163-B29] Svihus B. , Zimonja O. 2011. Chemical alterations with nutritional consequences due to pelleting animal feeds: a review. Anim. Prod. Sci. 51:590–596. 10.1071/AN11004

[txaf163-B30] Thomas M. , van der Poel A. F. B. 2020. Fundamental factors in feed manufacturing: towards a unifying conditioning/pelleting framework. Anim. Feed Sci. Technol. 268:114612. 10.1016/j.anifeedsci.2020.114612

[txaf163-B31] Thorpe S. R. , Baynes J. W. 2003. Maillard reaction products in tissue proteins: new products and perspectives. Amino Acids. 25:275–281. 10.1007/s00726-003-0017-9 14661090

[txaf163-B32] Truelock C. N. , Tokach M. D., Stark C. R., Paulk C. B. 2020. Pelleting and starch characteristics of diets containing different corn varieties. Transl. Anim. Sci. 4:txaa189. 10.1093/tas/txaa189 33241191 PMC7680177

[txaf163-B33] Truelock C. N. , Yoder A. D., Evans C. E., Stark C. R., Paulk C. B. 2022. The effects of pelleting process parameters and phytase source on the in-feed stability of phytase. Anim. Feed Sci. Technol. 294:115407. 10.1016/j.anifeedsci.2022.115407

[txaf163-B22502754] Vukmirović Đ. , R. Colovic, S. Rakita, T. Brlek, O. Duragic, and D. Sola-Oriol. 2017. Importance of feed structure (particle size) AND feed form (mash vs. pellets) in pig nutrition – A review. Anim. Feed Sci. Technol. 233:133–144. 10.1016/j.anifeedsci.2017.06.016

